# Retraction: P38 and ERK1/2 MAPKs Act in Opposition to Regulate BMP9-Induced Osteogenic Differentiation of Mesenchymal Progenitor Cells

**DOI:** 10.1371/journal.pone.0207157

**Published:** 2018-11-02

**Authors:** The PLOS ONE Editors

After publication of this article [1], concerns were raised about similarities between the following figures in the *PLOS ONE* article and an article published earlier by the same corresponding author in *BMB Reports* [2]. The two articles reported some differences in experimental sample labeling or treatment conditions, as noted.

- Fig 1A [1] and Fig 1C [2]: Except that Smad 1/5/8 data are different, calling into question whether the appropriate experimental control (Smad 1/5/8 blot control for the pSmad experiment) is shown in each case.
- Fig 2A and 2B [1] and Fig 3A and 3B [2]: Different statistical results indicated in the two articles by asterisks.
- Fig 4A [1] and Fig 3C [2]: C3H10T1/2 panels for BMP9, BMP9+SB, BMP9+PD panels. Different concentrations of SB and PD provided in the figure legends, with concentrations swapped for the two drugs. The Fig 4 legend and panel labels note the use of different compounds in [1]: the figure legend refers to SB203580 whereas the panel labels refer to SB231580. SB203580 was reportedly used in [2].
- Fig 5A [1] and Fig 3D [2]: The same experiment is reported, though different blots are provided.
- Fig 5B [1] and Fig 3E [2]: The figure legends in the two articles noted different concentrations of SB and PD (concentrations were swapped for the two drugs), and different timepoint information (2 hrs versus 4 hrs). The BMP9 + SB panels are the same in the two articles, but the PD panel in [1] is noted as “Blank” in [2].
- Fig 5C [1] and Fig 3F [2]
- Fig 6A [1] and Fig 4A [2]: Both blots in upper panel and b-actin blots in the lower panel.
- Fig 6B [1] and Fig 4B [2]
- Fig 6C [1] and Fig 4C [2]: All panels appear duplicated except that BMP9 and BMP9+NC panels have labels switched in the two articles.

Overall, the two articles address the same research question and report many of the same experiments, although there are some experiments uniquely reported in each article. There is also substantial text overlap in the articles, including in the Abstract and Introduction. The *BMB Reports* article was not cited in the *PLOS ONE* article.

When queried about the figure similarities, the corresponding author noted that the original data underlying the *PLOS ONE* article are no longer available and requested retraction.

In light of the concerns about redundant publication of data in several figures, the unavailability of the underlying data, and questions as to the accuracy with which experimental results were reported in some cases, the *PLOS ONE* Editors retract this article.

The authors did not comment on the retraction decision.

## References

[1] Zhao Y, Song T, Wang W, Wang J, He J, Wu N, et al (2012) P38 and ERK1/2 MAPKs Act in Opposition to Regulate BMP9-Induced Osteogenic Differentiation of Mesenchymal Progenitor Cells. PLoS ONE 7(8): e43383 10.1371/journal.pone.0043383 22912865 PMC3422272

[2] Xu D-J, Zhao Y-Z, Wang J, He J-W, Weng Y-G, Luo J-Y. (2012). Smads, p38 and ERK1/2 are involved in BMP9-induced osteogenic differentiation of C3H10T1/2 mesenchymal stem cells. BMB Reports 45(4): 247–252. 22531136 10.5483/bmbrep.2012.45.4.247

